# Eureka!: What Is Innovation, How Does It Develop, and Who Does It?

**DOI:** 10.1111/cdev.12549

**Published:** 2016-05-31

**Authors:** Kayleigh Carr, Rachel L. Kendal, Emma G. Flynn

**Affiliations:** ^1^ Durham University

## Abstract

Innovation is not only central to changes in traditional practice but arguably responsible for humanity's remarkable success at colonizing the earth and diversifying the products, technologies, and systems within it. Surprisingly little is known of how this integral component of behavioral flexibility develops and the factors that are responsible for individual differences therein. This review highlights two primary ways in which the process and development of innovation may be better understood: By emulating the critical advances of animal behavior researchers in examining innovation in nonhuman species and establishing a clearer conceptualization of what is “innovation”. A pathway to innovation is suggested and an innovation classification system offered to aid recognition of its appearance and potential cultural contributions.

Around 70,000–80,000 years ago, in the African Middle Stone Age, technological and behavioral innovations suggestive of modern human capacities appeared (Mellars, 2005). Although the widespread emergence of complex human culture is typically ascribed to the later developments of the European Upper Paleolithic (Shennan, 2001), there is little doubt that humanity's creative revolution sparked some tens of thousands of years ago (long after the earliest displays of hominin tool use, estimated 2.6–1.4 Ma; see Nielsen, 2012).

Advances in human cognition over evolutionary history have engendered inventions and innovations of such sophistication, and complexity, that they surpass those of all other nonhuman species. Whether fueled by one factor or a combination, including brain evolution, demography or social network size, climate change, emergence of language and cooperation (e.g., Elias, 2012), it is irrefutable that humans have creatively and culturally excelled. Given its critical importance to our success, it is surprising that our understanding of innovation in humans, including its evolutionary foundations, developmental trajectory, and contextual facilitators, is still in its infancy. As such, developmental psychologists have much to contribute to the innovation discussion and much to gain.

## Innovation

### Placing Developmental Psychology on the Stage of Innovation Research

There is a rich history, across academic disciplines, of applying the concepts, theories, and empirical advances of one field of study to another. Here, we aim to draw together knowledge from comparative psychology, developmental psychology, and animal behavior research to contend, as Want and Harris (2002) did in relation to the social learning of tool use, that the much needed dedicated developmental study of innovation may be informed and accelerated by an analysis of research elsewhere. Although this analysis needs to be applied to all aspects of study, including research questions, techniques, tasks, and findings, here our primary goal is developing an agreed‐upon definition. An essential first step in advancing our understanding of innovation is determining precisely what is meant by this term. A clear definition will aid in decisions about (a) who we conceptualize as innovators, (b) the form of behavior labeled as innovation, (c) the frequency of innovation, and (d) the contribution innovation makes to cumulative culture (a major discussion point in the ensuing sections). The current lack of operationalization within developmental psychology, in contrast to work within the animal behavior field, may be impeding research progress by preventing the establishment of a common “language” with which to discuss innovation, a language that carefully separates innovation from related yet conceptually and cognitively distinct constructs, uses similar terminology and criteria for identification (depending on the “form” that it takes; see Markers of Childhood Innovation, Point 1), and resists human centricity such that comparisons with other species can be made. Achieving greater consistency in terminology use, by delineating terms associated but not synonymous with innovation and increasing collaboration between developmental and comparative researchers, is therefore imperative.

Childhood innovations appear in a number of domains: games, pretend play, drawing, storytelling, and more general language. In this article, we focus on *behavioral* innovation in the physical domain, specifically novel problem solving in the context of tool use. We do so for several reasons. First, novel objects, in the form of artifacts and tools, saturate our world, and we must understand and use an enormous array of them from a very early age. If “learning to use tools and artifacts is inextricably linked to the developmental study of *imitation*” (Carpenter & Nielsen, 2008, p. 225; emphasis added), then their invention or modification is inextricably linked to the developmental study of *innovation*. Second, in pursuing a working definition for developmental psychologists, we are mindful of the need for innovation to be a “useful and usable concept” (Reader & Laland, 2003, p. 11); that is, the concept affords transparency in meaning and is one with which researchers can theoretically and experimentally engage. An overarching definition is desirable, but how the innovation phenomenon is expressed between domains may be diverse. Hence, a narrowing of focus to the physical domain is necessary in this case. Finally, there is a wealth of tool innovation research with nonhuman animals from which knowledge may be drawn and critical cross‐species comparisons made. This aids understanding of the phylogenetic (evolutionary) development of innovation and helps uncover phylogenetic relationships, uniqueness and origins of abilities, the influence of culture, language, and so on.

An important question to address is why it is necessary to bring questions about innovation to the developmental field. Crucially, compared to research on social learning (e.g., special issues in the *Journal of Experimental Child Psychology*, 2008; *Philosophical Transactions of the Royal Society B*., 2009; *Developmental Psychology*, 2013), the development of innovation in humans has received little attention. However, innovation and social learning may be regarded as two sides of the same coin, closely related in terms of their likely underlying mechanisms (Heyes, 2012) and their complementary roles in the acquisition, transmission, and evolution of culture, meaning insights into one will be highly informative for the other. Furthermore, adaptive trade‐offs operate between the two (Kendal, Coe, & Laland, 2005) such that observing when children innovate will help reveal the conditions under which they judge *imitation* a comparatively less effective learning strategy (addressing a “why” question of innovation). It makes little sense, therefore, to know so much about one side of the coin (social learning) and so little about the other (innovation).

In general, observations of “innovative” behavior (in the sense of noncopying) within the social learning literature have largely been treated as secondary or anomalous findings and thus not pursued. The lack of innovation research may be due to the rarity with which children deviate from social information in experimental contexts and, in turn, produce novel behavior. This is compounded by the lack of opportunity for innovation in social learning studies given they are not designed to afford this. Importantly, infrequency does not equate to incapability. Furthering our understanding of how, and why, innovation operates ontogenetically (develops over time in an individual) is essential to understand its typical trajectory, behavioral manifestations, mechanisms, relations with other aspects of cognition (constituent processes such as exploration, play, tool use, and problem solving in the case of physical cognition), individual differences in “innovativeness”, and ultimately how it may be enhanced (see also Chappell et al., 2015). Comparing innovative propensities across age groups will prove fundamental to establishing the developmental factors that impact upon innovation across the life span. Developmental changes in imitation (including “overimitation”), normativity, functional fixedness, and cognitive flexibility are such potential influencing factors.

First, we reflect on the importance of innovation from the wider perspective of cultural evolution, demonstrating the need for a deeper understanding from developmental psychology of the development of and requirements for innovation. In the “Identifying Innovations” section, we draw upon theoretical and nonhuman animal research to present an overview of the requirements for innovation, and construct a theoretical pathway to innovation. In the “Theoretical Contributions” section, we formulate an operational definition of innovation and an accompanying classification system. We close in the “Conclusion and Future Directions” section by proposing future avenues for research.

### The Cultural and Evolutionary Importance of Innovation

Cultural innovation is to cultural evolution what mutation is to biological evolution: without innovation, cultural traits and therefore cultural transmission would not exist. [Lehmann, Feldman, and Kaeuffer, 2010, p. 2356]

Biologists Lehmann, Feldman, and Kaeuffer (2010, p. 2356) perfectly summarize the critical nature of innovation within cultural evolution. Innovations, whether products, actions, or behavior, have not only aided in the generation of cultures (group‐typical behavior patterns, shared by members of [animal] communities, that are to some degree reliant on socially learned and transmitted information; Laland & Janik, 2006, p. 542) but also elaborate cultural systems wherein knowledge is repeatedly built upon and products and practices progressively modified and improved. The repeated modification of cultural traits, increasing the trait's complexity or efficiency, is the hallmark of a *cumulative* culture (Dean, Vale, Laland, Flynn, & Kendal, 2013). Technological innovations, in particular, are often not the output of any single individual but the result of collective and incremental efforts over time. Concepts, ideas, and discoveries of predecessors inform problems anew, such that designs may be honed, flaws corrected, and efficiency increased. Such “cultural ratcheting” (Tennie, Call, & Tomasello, 2009) would not be possible in the absence of high‐fidelity social learning (e.g., imitation, innovation's cultural counterpart), enabling the intergenerational preservation of knowledge and the transmission of innovated modifications (Boyd & Richerson, 1985). These processes are intricately entwined; indeed, “the transmission process itself can be a continuous creator of innovation” (O'Brien & Shennan, 2010, p. 8). Together, innovation and high‐fidelity transmission establish traditions, enable cultural products to proliferate and evolve, and are likely candidates in the search for what makes our species, and our capacity for cumulative culture, so unique (Dean et al., 2013).

The above is, of course, an oversimplification of the development and maintenance of cultural systems, insofar as not all innovations are “good” (i.e., solve problems or increase efficiency) nor are all “good” innovations adopted. It is beyond the scope of this article to unpack the complexities of how cultural systems evolve, but we acknowledge that change will not inevitably ratchet “up” sophistication and efficiency (of a technology or behavior). Moreover, as different cultural traits enjoy different levels of success and longevity, considering *resistance* to innovations is just as important as their adoption and transmission.

Although this review ultimately provides an individual‐level definition of innovation, it is impossible to detach discussion of individual innovations from discussion of cultural innovations. This is because when assessing the impact or adaptive value of an innovation, it is more difficult (and subjective) when that innovation belongs to a sole individual. What may be adaptive to one individual may be non‐ or maladaptive to another, depending on one's criteria. Certainly, the value of an individual innovation is easier to infer when its usefulness or efficiency is readily apparent. However, a more objective measure of an innovation's adaptive value, or capacity to induce change, is the degree to which it is a cultural innovation in being transmitted to other individuals (see Markers of Childhood Innovation, Point 6).

In theory, the adaptive benefits of an individual‐level innovation may be vast. To innovate is to potentially maximize exploitable resources, increase the efficacy of one's behavior, and circumvent novel challenges and threats. By allowing individuals to better adapt and respond to changing environments, innovation maximizes survival. In a positive feedback loop, novel behavior favors more able individuals, creating selection pressures for brain areas responsible for complex technical behavior (Reader & Laland, 2002) and, in turn, favoring the emergence of yet more complex behavior. Indeed, greater numbers and diversity of technical innovations are implicated in the evolution of brain size in birds (Overington, Morand‐Ferron, Boogert, & Lefebvre, 2009) and primates (Reader, Hager, & Laland, 2011). As with social learning, however, there are costs to the indiscriminate use of a learning strategy. Innovation must be considered most adaptive when flexibly utilized (Toelch, Bruce, Meeus, & Reader, 2011). Moreover, deviating from established behavior is inherently risky, meaning “a certain level of hesitancy to adopting novel behaviors is warranted” (Brosnan & Hopper, 2014, p. 1).

## Identifying Innovations

Innovation definitions and delineations from the animal behavior field have abounded in recent years. Reader and Laland's (2003) comprehensive appraisal of the animal innovation literature formulated two widely cited definitions of the phenomenon: (a) An innovation (sensu product) is a new or modified learned behavior not previously found in the population, and (b) innovation (sensu process) results in new or modified learned behavior and introduces novel behavioral variants into a population's repertoire. Although there is no surer way of determining innovation than if it has never before been seen in a population, this definition raised the expectation of long‐term monitoring in order to observe behavioral origins (which, although challenging, some have met; van Schaik, van Noordwijk, & Wich, 2006). Ramsey, Bastian, and van Schaik (2007) conversely endorsed the view that “Innovation is the process that generates *in an individual* a novel learned behavior” (p. 393, emphasis added). Determining the level at which to pitch innovation for developmental research is one of several reasons why it would not be appropriate to simply adopt existing definitions. As with applying the particular methods of animal behavior researchers, it is important to consider how requirements for innovation translate between species.

To fully understand the evolution, development, consistency, and extensiveness of children's innovation, a clear definition, workable across a variety of contexts, is needed. The shortage of developmental work necessitates that, in our journey toward a definition, we reflect upon alternative bodies of literature (including animal and human adult). However, the focus remains on its relevance and applicability to childhood and development.

### Markers of Childhood Innovation

Childhood is a time of exploration, play, and learning. The potential to discover and produce unusual or novel behavior is vast. Are each of these occurrences to be considered an innovation? We think not. There are criteria which a potential innovation must meet, and this forms the basis of both the ensuing discussion and our innovation definition (see Theoretical Contributions).

1. Innovation can be the result of asocial learning or a combination of asocial and social learning, but it must be novel.

At the uppermost level of distinction, learning may be social (information is acquired from others), asocial/individual (independent of social observation or interaction), or a combination of the two. Although innovation may be considered “largely asocial learning” (Kendal, Giraldeau, & Laland, 2009, p. 218), in that the innovator ultimately produces behavior that has not, in its full form, been socially observed, it is often an evaluation of information acquired socially that induces innovation; specifically, judging “that a novel solution to a problem generates superior returns than does an established (observed) behaviour” (Laland, 2004, p. 10, parentheses added). It is not, therefore, technically independent of any social influence. This leads us to our proposition that innovation is not wholly asocial (nor, indeed, is all asocial learning innovation). Thus, it is advantageous to assign beneath the “innovation” umbrella the terms of *independent invention*, when novel behavior results from asocial learning, and *modification*, when social influences are directly implicated (as in cumulative culture). There are two main reasons why we believe this distinction to be advantageous, both of which are revisited later in this section. First, the two forms may have different cognitive underpinnings and different developmental profiles, meaning inferences or generalizations about children's abilities cannot be made on the basis of the assessment of only one form. Second, they likely contribute differently to processes of cumulative culture and cultural transmission, partially as a result of the primary source of information from which they draw.

Note that in the case of independent invention, we do not refute that individuals will be equipped with some social information acquired from prior interactions and experiences with the world (e.g., in inventing a novel tool, the components that make up the tool may not themselves be novel), including products of others’ behavior. Rather, what we aim to distinguish is whether asocial learning is the predominant learning mechanism involved in producing the innovation (there is no immediate social learning from which the impetus for the innovation directly emerges, as with innovation by modification). Making this distinction will not always be straightforward and indeed becomes blurred when “goal emulation,” where the means of achieving a socially observed goal is arrived at through a different means, may be considered innovation by invention or modification (see Point 5). Nevertheless, the idea that independent invention should be regarded as one form of innovation, that is, a clear derivative of asocial/individual learning, has theoretical support (e.g., Kandler & Laland, 2009; Lewis & Laland, 2012; Slater & Lachlan, 2003).

Human tool use is, in its frequency, flexibility, and complexity, unique within the animal kingdom (Kacelnik, 2009). Tool‐use learning has been extensively investigated in social learning paradigms, designed to understand the age at which children become proficient tool users, the factors that enable it, and the cognitive systems that differentiate humans from nonhumans. Tool‐use learning has similar potential to inform and direct investigations of children's innovation. Although few in number, examinations of tool‐use innovation in children have revealed one consistent finding: Children are poor innovators. Hanus, Mendes, Tennie, and Call (2011) compared apes and human children in a “floating peanut” task in which water had to be used as a tool to retrieve a peanut from the bottom of a narrow tube. The developmental progression in children's success was marked, with only 8% of 4‐year‐olds, but 58% of 8‐year‐olds succeeding. The authors attributed this to the greater cognitive flexibility of the older children, facilitating their abandonment of ineffective methods, together with their enhanced exploration, insight, and attention to alternative task components. Nielsen (2013) replicated the finding that 4‐year‐olds experience great difficulty producing the necessary innovative behavior in the floating object task yet acquire the solution immediately following the demonstration of a knowledgeable adult. Hence, the problem is not one of performance but identification and generation of the required response.

In a similar reflection on comparative literature, Beck, Apperly, Chappell, Guthrie, and Cutting (2011) presented 3‐ to 11‐year‐old children with a task originally used with New Caledonian crows. The task required manufacturing a novel tool (a hook from a pipe cleaner) to extract a bucket from a tube. As in Nielsen's study, tool innovation was difficult for the youngest children and success increased with age. Task variations including tool preference selection and prior object manipulation did not impact upon performance. A social demonstration, however, permitted nearly all children to succeed. The “ill‐structured” nature of tool innovation problems was offered as an account for the findings, with the absence of clearly defined strategies for moving between the starting conditions and goal states theorized to impede progress. A recent study employing the same task to compare Western and Bushman children, aged between 3 and 5 years, further suggests that cognitive limitations underlie innovation difficulties (Nielsen, Tomaselli, Mushin, & Whiten, 2014). Somewhat surprisingly, despite vast differences in cultural environments and exposure to premade artifacts, both groups evidenced similarly poor tool innovation. Further research into how the capacity for innovation emerges (precisely which cognitive factors are implicated) will only be possible by continuing developmental investigations of this kind.

According to our delineation, these studies examine innovation by independent invention but not innovation by modification. Their importance cannot be disputed: Novel problem‐solving tasks offer a highly suitable means to reflect upon children's capacity for novel invention. Asocial control participants of social learning studies offer similar insight. The invention–modification distinction may not be universally accepted as a necessary one, but we nonetheless believe it has utility. There are reasons to believe that the two forms of innovation will have different primary difficulties associated with them, potentially altering their developmental profile. Although the ill‐structured nature of problems proves challenging for novel invention tasks, an ontogenetic imitation bias induced by social information (e.g., Horner & Whiten, 2005) is highly likely to prove equally challenging for modification tasks by impacting the generation of alternate *asocial* output (Wood, Kendal, & Flynn, 2013).

With regard to capacities for cumulative culture, tasks must permit opportunities for modification, refinement, and/or recombination of established behavior in order to mirror the ratcheting process. One serendipitous, but influential, invention may outweigh iterative alterations when it comes to cultural diversity (Kandler & Laland, 2009), but novel invention is of lesser consequence for cumulative culture (Lewis & Laland, 2012). These theoretical findings support the deconstruction of innovation (for both definition and study), owing to the wider cultural implications of innovation's various forms.

Irrespective of the form it takes, the concept of innovation is tied to that of novelty (Reader & Laland, 2003). Given that we already have opposing views of population‐ and individual‐level novelty in the animal literature, how is novelty to be judged? In experimental research, by introducing novel tasks we are able to say that any behavior exhibited that has not previously been socially observed (in its full form) is indeed new *to that individual*. Where tasks are posed in group contexts, the first “solver” meets the population‐level definition of an innovator (producing behavior not previously found in the “population”), along with any individual who introduces a new solution (whether completely new or a combination or modification of observed behavior; Flynn & Whiten, 2012; Whiten & Flynn, 2010). Because almost every new behavior resembles, if not contains, existing behavioral constituents, a strict definition of novelty would be unwise. In the animal literature (following Kummer & Goodall, 1985), innovation is additionally assessed in light of the *context* in which the behavior is performed. Thus, either the innovation‐inducing problem (necessitating use of novel or existing behavior patterns) or the solution to an existing problem may be novel (again, without the basic behavioral and motor elements necessarily being so).

There are a number of hypothesized contributors or precursors to the innovation process. These include, but are not limited to, causal understanding, insight, curiosity, exploration (discovery learning), divergent thinking, and creativity. They do not equate to innovation and alone are not sufficient to produce it.

Just as imitation, emulation, mimicry, and enhancement learning possess commonalities, requiring specific experimental designs to delineate them, so innovation shares elements of its process and product with other related constructs. Particularly in their combination, these constructs facilitate higher level cognition thereby promoting the cognitive maturation plausibly conducive to innovation.

*Causal understanding* denotes an appreciation of the causal relation underpinning a covariance. Knowing what causes something means knowing how it may be changed, and this is central to humanity's technological achievements (Vaesen, 2012). Deducing causal understanding from the production of an innovation is met with caution by some animal researchers, particularly when innovative problem solving “may be more parsimoniously explained by simple, conserved associative processes” (Thornton & Samson, 2012, p. 1466). Causal knowledge does, however, play an important role in human ontogeny, and specifically cognitive development. Within the first 2 years of life, causal learning is evident in children's interpretations of events (Walker & Gopnik, 2014) and by the 5th year, causal‐based inductions direct children's category‐based reasoning (Hayes & Thompson, 2007). While simple causal understanding does not require high‐level reasoning abilities, it may be that the latter better facilitates the innovation process. It is also necessary to consider the relation between task difficulty and causal understanding development: Younger children may have sufficient causal knowledge to innovate on simpler tasks but not more complex ones. Flexible inductive reasoning, wherein a variety of inferences may be made about a single item that fits multiple categories, develops throughout childhood (e.g., Bright & Feeney, 2014). Such sophisticated reasoning, involving the consideration of multiple possible outcomes, may allow children to better evaluate the employment of social and/or asocial information and consequently utilize innovation when it is most appropriate.

*Insight*, defined as “the sudden production of new adaptive responses not arrived at by trial behavior… or the solution to a problem by the sudden adaptive reorganization of experience” (Thorpe, 1964, p. 110), may also play a role in the innovation process. We note, however, that if one accepts innovations that need not possess intentionality (Point 7) and may arise accidentally, insight need not be implicated. For Kacelnik (2009, p. 10072), “Even in humans, the causal use of the term insight is ridden with difficulties, and it can hardly be claimed to explain much.” We remain uncertain regarding how much emphasis should be placed upon insight; it clearly has some role in certain forms of novel behavior but does not encapsulate all instances of novel problem solving and, further, is very difficult to determine.

Outwardly, *curiosity* appears a more neutral and less contested term to impart. It captures an individual's motivation to discover and learn more about the environment. Being curious acts to prompt exploration. Yet, as with insight, there is also the implication of foresight (Hauser, 2003): a reason to be curious in the first place (“what does this do, and why?”). There obviously exist objects that promote curiosity, a prime example of which are artificial fruit tasks, widely used by developmental psychologists and in animal behavior research (e.g., Horner & Whiten, 2005). They contain the motivation (food reward) for animals to interact with and explore artifacts.

A concept closely tied to curiosity is *exploration*; clearly, trying to work out ways in which to do something differently requires exploratory testing of ideas, paving the way for innovation (Sol, Griffin, Bartomeus, & Boyce, 2011). Remarkable advances have been made in understanding how children's exploratory play allows them to formulate theories of, and learn about, the world. In using play to test hypotheses and generate causal knowledge, they may be viewed as “like” scientists (Gopnik, 2012). Importantly, play provides children with information about the functionality of objects that, if not immediately relevant, may have future use. Animals also play, but the key difference for our own species is pretense. Pretense as a specific form of play, wherein individuals generate and reason with imagined (often novel) scenarios and objects, has been touted as a springboard for innovation through its promotion of creativity (Nielsen, 2012; Picciuto & Carruthers, 2012). The evolutionary function of pretend play is, indeed, considered to be the practice of creative thought (Carruthers, 2002).

Exploration, whether inside or outside of an imagined setting, can promote new learning, contributing to an appreciation of how behaving in a novel manner may yield different, perhaps more efficient outcomes. The significance of age in a discussion of innovative tendencies is tied to the question of how much an individual may benefit from greater, and more diverse, exploration. That is, recognizing that a new response is required and physically producing one may require the competence and experience of adulthood (Kendal, Coe, & Laland 2005). Though certainly related, exploration is *qualitatively distinct* to innovation (Reader & Laland, 2003): You may explore, but you may not always innovate.

A number of predictions may be made regarding the interplay between exploration and familiarity (or expertise) in a given domain. Exploration is certainly likely to increase familiarity, but what of the reverse effect? Simonton (2000) notes, when considering creative achievement, that domain‐relevant experiences are of importance. Although there is variability and a number of factors that feed into the relation, it appears that cumulative experience within a domain enhances creative impact. There is, therefore, an argument to be made that familiarity will prompt more directed exploration and increase the likelihood of innovation production. However, the nature of prior experience in a domain will viably make it more or less likely that an individual is motivated to explore. They may be less willing to consider alternative behavior if their prior experiences are associated with some negative consequence. Moreover, familiarity can also heighten functional fixedness and conservatism (Point 3).

Exploration is particularly potent in its combination with *divergent thinking* (essentially the opposite of functional fixedness). Divergent thinking denotes the ability to search for new ideas (Guildford, 1959, as cited in Bijvoet‐van den Berg & Hoicka, 2014) and is thus implicated not only in problem solving but in creative potential and productivity (Runco & Acar, 2012). Even at 2 years of age, children demonstrate individual differences in divergent thinking, with evidence to suggest that greater exploration (producing a variety of actions on a novel object) is linked with originality (Bijvoet‐van den Berg & Hoicka, 2014). While originality links divergent thinking and creativity (and innovation), they are not synonymous; one can demonstrate good divergent thinking without demonstrating creativity (Runco & Acar, 2012). Conceiving multiple potential solutions to a puzzle does not imply they will be good, useful, or workable (Runco & Acar, 2012). In contrast, typical definitions of *creativity* require that creative ideas, behavior, and problem solving be both original *and* valuable (Picciuto & Carruthers, 2012). One's perspective regarding *to whom* these must be valuable inevitably alters the goalposts of creativity.

Many relevant ideas regarding the innovation–creativity distinction are offered by Levitt (1963). Principally, creative thoughts are regarded as a precipitating factor for innovation but must undergo conversion to qualify as such. It is the difference between generating ideas and implementing them: the abstract versus the concrete. This is a common distinction made in business, but one that is consistent with Simonton's (2003, p. 311) conceptualization of innovation as “the end product of a creative process.”

**Figure 1:**
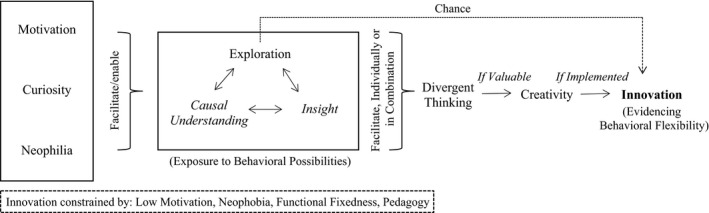
A hypothetical individual‐level pathway to innovation. Arrows denote which construct leads to another construct. From left to right, any of the processes within the first block can lead to those within the second block. The constructs in italic text within the second block play more contested, or less direct, roles in this pathway (see Point 2). Neophilia, and its opposing construct neophobia, are discussed in Point 3. Context and prior learning (social and/or asocial) are acknowledged to potentially contribute to each construct portrayed and to differentially promote behavioral change. Innovation is generally regarded as a component of behavioral flexibility by allowing “individuals to react to environmental changes… [by] changing established behavior” (Toelch et al., 2011, p. 1). It should be noted that, rather than necessarily prompting divergent thinking and creativity, exploration may allow an individual to stumble upon an innovation by chance, captured by the connecting arrow.

From this discussion, we have formulated a hypothetical pathway to innovation (Figure 1). We tentatively offer this pathway as a starting point, with the hope that it will stimulate debate and be improved upon by subsequent research. By presenting the precursors to innovation, we also hope it may serve as a useful theoretical framework for educators, and individuals in various sectors, who wish to consider ways to promote the innovation process.

Functional fixedness (conservatism), low motivation, pedagogy, and neophobia restrict innovation.

*Functional fixedness*, or behavioral conservatism, is a likely inhibitory factor in innovation. It denotes fixation upon the demonstrated or learned design function of an object as the proper, conventional, or normative way to use it. Children attain such a concept of artifact function at around 6 or 7 years of age (Defeyter & German, 2003), prior to which time they ostensibly possess greater flexibility in artifact use. The development of functional fixedness impacts innovation: Categorizing an object as “for” a particular function means using it in a way not initially intended by its design, as is often required in tool innovation and novel problem solving, is difficult and serves to compound the imitation bias. Younger children may be more “immune” to functional fixedness (it affects 7‐year‐olds to a greater extent than 5‐year‐olds; Defeyter, Avons, & German, 2007) but disadvantaged by more general cognitive immaturity. Discovering ways to reduce its effects will plausibly enhance children's developing capacity for innovation. Due to its combination with artifacts, functional fixedness is a unique problem when studying innovation within the context of tool use. Investigations outside of this domain will only prove complementary and extend our understanding of when and why children experience difficulties.

As expected, *motivation* is closely tied to innovation propensity (Reader & Laland, 2003). Whether arising from factors in the environment, such as a food reward, or from a stable individual motivational component to discover more (Sol, Griffin, & Bartomeus, 2012), it can be viewed as a necessary starting constituent of the innovation process, prompting exploration. The understanding and knowledge that can be ascertained through exploring the environment makes *pedagogy* (explicit direction or teaching) a “double‐edged sword”: In the same way as observation (Wood et al., 2013), it leads to efficient, but restricted, exploration and learning in preschoolers (Bonawitz et al., 2011).

Open diffusion studies, involving the introduction of a model and task to a group of freely interacting novices, have provided opportunities to reflect upon biographic, social, cognitive, and temperament predictors of social learning (Flynn & Whiten, 2012). Specifically, increasing age, popularity, dominance, and impulsivity have been seen to promote children's successful interactions with a foraging apparatus. Animal studies have found the predictors of *innovation* (here, successful novel problem solving or foraging) to include exploration, neophilia—being novelty inclined or unafraid to approach or interact with new objects—and persistence (e.g., Benson‐Amram & Holekamp, 2012; Thornton & Samson, 2012). *Neophobia*, the fear of novelty, conversely acts to restrict exploration intensity (Sol et al., 2012) and thereby plausibly innovation. Certain social factors, such as the presence of conspecifics (Griffin, Lermite, Perea, & Guez, 2013), appear to similarly deter innovative foraging in animals. This latter research demonstrates the need to consider extrinsic, as well as intrinsic, influences on the expression of innovation. Given the heightened social motivations of children, and humans more generally, social and contextual factors will have a large role to play in an individual's decision to deviate from established behavior.

Innovations, being of multiple origins, may be cognitively distinguished.

A number of potential sources of innovation have been identified, all of which are deemed capable of introducing new cultural variation into a population. These include, as listed by Mesoudi et al. (2013), chance factors (i.e., accidents and copying errors), novel invention (be it through trial and error, insight, or exploration), refinement (modification and improvement), recombination (of behavioral variants), and exaptation (the application of behavior to a new function). The implication is that innovations are not equal: Although the endproducts may look remarkably similar, the processes from which they have arisen may differ. What is important for innovation classification is that, in each case, independent of source, the behavioral outcome is recognized as viable and useful. These judgments will not be free of subjectivity.

Recognition of innovation sources has led to the categorization of “types” of innovation, potentially impacting upon their study and measurement. In accordance with Ramsey et al. (2007) who endorse a “cognitively simple” and “cognitively complex” innovation distinction, Rendell, Hoppitt, and Kendal (2007) refer to “passive” and “active” innovations with the former in both cases involving chance factors. In a study examining the social learning and innovative propensities of common marmosets, Burkart, Strasser, and Foglia (2009) offered a similar operationalization; Type I innovations correspond closely to common conceptions of innovation involving goal‐directed and problem‐induced behavior, and Type II innovations, in contrast, are characterized as more incidental, and plausibly accidental, arising not due to the need for a solution to a problem but as a result of situations offering chance, and scope for, novel behaviors. Thus, authors include an idea of weak and strong innovations, the latter denoting active “thinking up” of novel behavior and resonating with typical definitions of fluid intelligence (including the ability to solve novel problems). The developmental trajectories of these two types of innovations may be distinct. One could hypothesize that Type I (“active” innovations) will be more prevalent in late rather than early childhood, when individuals are equipped with greater experience and cognitive maturity. However, as we discuss in relation to intentionality (see Point 7, and Theoretical Contributions), we believe the emphasis should be more upon subsequent learning.

For some, there is no value in identifying the origin of an innovation; the “ecological and evolutionary consequences of innovation need not depend on the cognitive sophistication of the innovative process” (Laland & Reader, 2010, p. 41). It may not be so much genius that underpins innovation as chance (Lewis & Laland, 2012). However, where an innovation occurs by chance, it may not be learned, thus not repeated and consequently neither useful nor influential in terms of cultural transmission and traditions (Reader & Laland, 2003).

Goal emulation can represent a weak form of innovation.

Emulation involves learning about object properties, affordances, and causal relations (Want & Harris, 2002). Affordance learning, one form of emulation, may be observed in ghost control experiments wherein the movements of an apparatus are demonstrated via hidden mechanisms, in the absence of a live model or agent. By matching the ghost demonstration, individuals evidence learning about the affordances of the action(s) and the properties of an object (beyond simple object movement re‐enactment). In goal emulation, the observer reproduces the model's goal but uses their own method (e.g., selecting a different tool). But what if, in this instance, the individual opts for an individually discovered *novel* method, involving the use of a novel tool for example? It may not be novel problem solving, but it *is* finding “a new solution to an old problem.” Whether this new solution is discovered by way of asocial learning, or a combination of asocial and social learning, dictates its designation as innovation by invention or innovation by modification (Point 1). In Cutting, Apperly, Chappell, and Beck (2014), children were shown a readymade pipe cleaner hook if they failed to solve the hook‐making task (described in Point 1). This may be regarded as both innovation by invention (despite having social information in the form of a premade hook, the social information itself is not being directly modified; rather, children are still required to invent the means by which to create the hook) and goal emulation (the socially observed goal is reproduced via the individual's own means).

The matter becomes more convoluted in the event that the goal being reproduced is one that does not solve the problem at hand, as “good” innovations should work (Hauser, 2003). Behavior that would otherwise be labeled “goal emulation” crosses into the boundary of “innovation” only when the novel modification of the preexisting behavior is useful and successful. When these criteria are not fulfilled, goal emulation indicates exploration and curiosity; an appreciation of alternative behavioral potentials when the cognitive capacity, motivation, or any factor reviewed earlier is not yet sufficient to enable the innovation process. In this way, goal emulation may be seen as a precursor to innovation in childhood (or a weak form of; see Whiten & Flynn, 2010, whose “innovate‐minor” category for children has the properties of emulation).

It is similarly pertinent to ask when the omission of actions within a behavioral sequence becomes an innovation, that is, a new modification. Goal emulation can involve such omissions, as in Horner and Whiten's (2005) comparative study. Although 3‐ to 4‐year‐old children imitated both causally relevant and irrelevant actions in a tool‐use task, irrespective of the availability of causal information (a transparent, but not opaque, puzzle box allowed the irrelevance of the actions to be seen), chimpanzees disregarded the irrelevant actions “in favor of a more efficient, emulative technique” (p. 164) when the box was transparent. Thus, the chimpanzee behavior became more efficient, but the goal itself, retrieval of a food reward, was not altered. Although apes emulate to a higher degree than children, their lack of faithful transmission mechanisms means more efficient behaviors are rarely acquired by others, resulting in an absence of cumulative culture (Dean et al., 2013). In summary, emulation and innovation by modification are differentiated by a change in goal: With innovation, the outcome of the behavior must be better or more efficient (e.g., retrieval of more food), whereas with emulation, the details of the behavior involved in order to reach that outcome increase in efficiency (e.g., fewer steps in the behavioral sequence).

An innovation should be useful and/or transmitted.

Although controversial, we believe many innovations are likely to be beneficial and adaptive for the individual and the population in the event of their successful social transmission. This complies with the human literature wherein there is the implication that innovations should represent an improvement upon current behavior (Caldwell & Millen, 2010) and allow us to formulate not only solutions to problems but increasingly effective and efficient ones, enabling culture to evolve (Dean et al., 2013; Tennie et al., 2009). A caveat to the view of “useful” innovations has emerged from studies of bird song, wherein innovations may be simply neutral in their fitness consequences as opposed to specifically adaptive or maladaptive (Slater & Lachlan, 2003). We can speculate that the same will be true of children's innovations, particularly if they arise in the context of play. What children define as useful may be very different from what we adults define as useful; it may be enjoyable, for example, as opposed to serving a practical purpose. Open diffusion studies (see Point 3), wherein deviation from established behavior is seen (Flynn & Whiten, 2012), are well placed to infer what children regard as useful and, in turn, what is transmitted.

Maladaptive behavior (inducing detrimental fitness consequences), however, also thrives within cultures. This may be because of indiscriminate copying, informational cascades, indirect transmission biases, copying errors, and the transfer of outdated information (Rendell et al., 2011), as opposed to the spread of “bad” innovations. The imitation of causally irrelevant actions and transmission of maladaptive information (by adults, Flynn & Smith, 2012; children, Horner & Whiten, 2005; guppies, Laland & Williams, 1998) demonstrates that a behavior pattern may be functionally ineffectual and yet still succeed in spreading to other individuals. Is this to suggest that, regardless of outward utility, novel behavioral displays be considered innovations if they are reproduced by other individuals? The answer is probably yes. By their act of transmission, the implication is that they are of *some* use. The guppies in Laland and Williams (1998) study may take a longer and energetically more costly route to a feeder when a shorter route is available, but in doing so they are able to remain within the safety of the shoal. Therefore, “usefulness” of behavior may not be immediately apparent and additional motivations to learn ostensibly maladaptive information must be considered. Mechanisms such as “adaptive filtering” (Enquist & Ghirlanda, 2007) provide a possible resolution to maladaptive cultural traits, contingent upon an individual's capacity to perceive and correctly identify behavioral consequences and make innovative modifications accordingly.

An innovation need not reflect intentionality, but it should lead to learning.

Views surrounding the intentionality of an innovation feed into discussions of innovation sources and types (see Point 4). Without the intention to act in a novel manner, we can assume subsequent production of innovative behavior results from chance factors. We believe the inclusion of intentionality as an innovation criterion for children is unrealistic and unnecessary. Our argument is threefold. First, the ability to plan behavior develops gradually throughout childhood. This is especially true of the more cognitively complex and flexible *advance* planning, requiring one to anticipate action outcomes, in which children do not show higher levels of proficiency until aged 9–10 years or older (Tecwyn, Thorpe, & Chappell, 2014). This notion of forward projection of outcomes bears resemblance to intentionality. Complex planning is not, of course, a facet of all innovative behavior, but it is a worthy consideration for more complex innovations nonetheless. Second, like insight, intentionality is difficult to determine. It is often indicated verbally when an intention is broken but arguably less so when it is met. Third, whether arising from accidental occurrence or intentionality, novelty impacts upon an individual's future behavior (and, by extension, in cumulative culture) when it is learned and repeated, both individually and more widely. We posit that an innovation technically remains an innovation but loses its value in the absence of its repetition and transmission.

For Ramsey et al. (2007) and Reader and Laland (2003), among others, an innovation can only be considered as such if it is accompanied by learning: if it becomes an established feature of behavior, or if affordance learning is evident prior to the discovery of the innovative act itself. With regard to experimental tasks, we cannot be sure that children have demonstrated a novel *learned* behavior in the absence of repeated trials with the same apparatus nor is there opportunity to infer its origin or source: Is it a purposeful behavior executed with prior intentionality, the result of a trial‐and‐error approach or a one‐off accident? It may also not be truly reflective of human culture to prescribe short time spans in which an innovation can occur. It should, however, be noted that learning is not universally considered to be essential to the innovation process, and a number of definitions do not require it. The matter of determining repetitions of innovative behavior in the wild is a particularly tricky one, as animals are not observed continuously. Yet, while “trivial and idiosyncratic one‐off” behavior (Reader & Laland, 2003, p. 11) is unlikely to be scientifically published (as an “interesting departure from established behavior”), actually observing an individual repeat a novel behavior is *explicitly* suggestive of its effectiveness and significance. In addition to repetition, further measures of learning include iterative reductions in latency to solve a task using the innovation and verbal self‐report. Ascribing intentionality to an innovation is therefore a minor issue compared to the more ultimate contribution that it may make to cultural evolution, should it be learned.

## Theoretical Contributions

### Proposed Definition

The following operational definition is offered, drawing from the analysis undertaken in this review:

In the physical realm, a behavioral innovation is a new, useful, and potentially transmitted learned behavior, arising from asocial learning (innovation by independent invention) or a combination of asocial and social learning (innovation by modification), that is produced so as to successfully solve a novel problem or an existing problem in a novel manner.

We wish to note that, although verifying the occurrence of learning is the ideal, it is not at this stage essential. This criterion has the potential to inhibit research due to innovation's rarity in early to middle childhood (Beck et al., 2011). Attempts to assess learning, whether via behavior repetition, task latency, or verbal self‐report, would nevertheless be both valuable and revealing, potentially uncovering age and individual differences in the extent to which it is evidenced by children.

### Classifying Innovations

Rather than assigning innovation “types” (Markers of Childhood Innovation, Point 4), we propose a classification system based upon levels (see Table 1). The aim is to remove some of the focus from the *source* of the innovation and allocate it instead to the *outcome*. Learning, here, becomes the key component and not whether the initial novel behavior is accidental or insightful. In both instances, with learning, the outcome may well be the same. Should an innovation ultimately become a cultural trait, by way of its successful transmission and acquisition by others, we may regard it as of a higher level than an innovation that remains in the repertoire of only one individual. By thinking about innovations in terms of their larger cultural contribution and population‐level consequences, we may achieve clearer discussion of their nature and avoid inconsistent use of terminology.

**Table 1:** Classifying Innovation

	Criteria (whether innovation by *invention* or *modification*)
Levels	
1: Low	Unlearned “chance” innovation not repeated by the individual
2: Mid	Individually learned innovation repeated by the individual
3: High	Individually learned innovation that is acquired by others
Types (from animal behavior)	
Cognitively simple/complex (Whiten & van Schaik, 2007)	Simple: An innovation that could arise by individual discovery. Complex: An innovation that requires causal inference and deliberate action; not likely to arise by accident.
Weak innovation/invention (Ramsey et al., 2007)	Weak innovation: An innovation in which social learning or environmental induction is implicated Invention: An innovation that is rarer, more novel, and involves more cognition.
Passive/active (Rendell et al., 2007)	Passive: An innovation that is more likely to rely on chance events. Active: An innovation that is more likely to reflect cognitive abilities of the innovator.
Type I/Type II (Burkart et al., 2009)	Type I: An innovation that is goal directed and problem induced. Type II: An innovation that is more incidental.

Note.

By presenting our “levels” and earlier literature's “types,” this table intends to highlight the increased clarity afforded by the former classification. Transition from mid‐ to high‐level innovation does not necessarily directly link to the “usefulness” of the innovation but may be a function of other social and contextual factors, such as the dependency of transmission on the identity of the innovator, due to directed social learning or transmission biases. Owing to its cultural transmission ramifications, learning is a key, and ideal, component of our levels criteria. However, it is not at this stage essential to demonstrate in child research given the difficulties of observing repetitions of innovative behavior.

## Conclusion and Future Directions

The pivotal role of innovation in behavioral change and cultural evolution has prompted much research interest from a wide variety of disciplines, but thus far it has been met with comparatively little attention from developmental psychologists. Its cognitive and cultural ramifications, and relevance to numerous contemporary contexts, including business enterprises, medical practices, education reforms, and climate change, underscore the imperative need to better understand the process and development of innovation. Throughout this article we have attempted to convey how emulating the advances of animal behavior research, and establishing a clear and consistent terminology, will be a crucial first step toward addressing this need and placing developmental psychology firmly on the stage of innovation research. In presenting a theoretical pathway to innovation and a new classification system, we also hope to stimulate interdisciplinary conversation and debate, encourage evidence‐based conceptual frameworks, and prompt further experimental work.

Our provision of innovation criteria is intended to promote and support future research in this domain. We note, however, that whether a criteria consensus is gained or not, criteria of *any* sort will be of no value should researchers not be explicit in their own decisions regarding what will and will not be accepted as instances of the phenomenon, and take steps to create tasks, and task contexts, reflective of these aims. If, for example, we contend that innovations should represent better or more efficient ways of achieving goals, then an arbitrary alteration of a task solution (i.e., turning a manipulandi left vs. right) reveals very little in this regard. Similarly, one task trial (i.e., attempts with a novel task) discloses little about an innovation's origin and cannot verify the occurrence of learning. Examining task solution alternation, only possible with the implementation of a number of response trials (Wood et al., 2013), is a promising way of comprehending imitative or innovative strategy use over time and, through the manipulation of other variables of interest, what is viably responsible for conservatism and flexibility in children's learning. The implementation of multiple experimental trials, and multiple “generations” of learners, will establish confidence in the findings of innovation (and innovation‐related) research.

As we face a host of environmental, social, and economic issues at a global level, taking steps to promote innovation will be key. Studies examining the ontogeny of tool innovation and the factors affecting age‐related competence are needed to uncover consistencies in how and when this capacity emerges, as well as research examining consistencies in the innovative tendencies of individuals, populations (i.e., cross‐cultural comparisons), and species. Such studies would allow for the identification of factors reliably implicated in observations of learning strategy variance and their systematic promotion. An appreciation of how competence interacts with motivational state, reward value, and social context will aid in the critical disentanglement of individual differences in innovative propensities. Without a better understanding of the innovation phenomenon, we cannot hope to truly understand humanity's uniqueness, cultural complexity, and future ability to adapt—nor our capacity to nurture and cultivate it.

## References

[cdev12549-bib-0001] Beck, S. R. , Apperly, I. A. , Chappell, J. , Guthrie, C. , & Cutting, N. (2011). Making tools isn't child's play. Cognition, 119, 301–306. doi:10.1016/j.cognition.2011.01.003 21315325 10.1016/j.cognition.2011.01.003

[cdev12549-bib-0002] Benson‐Amram, S. , & Holekamp, K. E. (2012). Innovative problem solving by wild spotted hyenas. Proceedings of the Royal Society B, 279, 4087–4095. doi:10.1098/rspb.2012.1450 22874748 10.1098/rspb.2012.1450 PMC3427591

[cdev12549-bib-0003] Bijvoet‐van den Berg, S. , & Hoicka, E. (2014). Individual differences and age‐related changes in divergent thinking in toddlers and preschoolers. Developmental Psychology, 50, 1629–1639. doi:10.1037/a0036131 24588519 10.1037/a0036131

[cdev12549-bib-0004] Bonawitz, E. , Shafto, P. , Gweon, H. , Goodman, N. D. , Spelke, E. , & Schulz, L. (2011). The double‐edged sword of pedagogy: Instruction limits spontaneous exploration and discovery. Cognition, 120, 322–330. doi:10.1016/j.cognition.2010.10.001 21216395 10.1016/j.cognition.2010.10.001 PMC3369499

[cdev12549-bib-0005] Boyd, R. , & Richerson, P. J. (1985). Culture and the evolutionary process. Chicago, IL: University of Chicago Press.

[cdev12549-bib-0006] Bright, A. K. , & Feeney, A. (2014). Causal knowledge and the development of inductive reasoning. Journal of Experimental Child Psychology, 122, 48–61. doi:10.1016/j.jecp.2013.11.015 24518051 10.1016/j.jecp.2013.11.015

[cdev12549-bib-0007] Brosnan, S. F. , & Hopper, L. M. (2014). Psychological limits on animal innovation. Animal Behaviour, 92, 325–332. doi:10.1016/j.anbehav.2014.02.026

[cdev12549-bib-0008] Burkart, J. M. , Strasser, A. , & Foglia, M. (2009). Trade‐offs between social learning and individual innovativeness in common marmosets, *Callithrix jacchus* . Animal Behaviour, 77, 1291–1301. doi:10.1016/j.anbehav.2009.02.006

[cdev12549-bib-0009] Caldwell, C. A. , & Millen, A. E. (2010). Conservatism in laboratory microsocieties: Unpredictable payoffs accentuate group‐specific traditions. Evolution and Human Behavior, 31, 123–130. doi:10.1016/j.evolhumbehav.2009.08.002

[cdev12549-bib-0010] Carpenter, M. , & Nielsen, M. (2008). Tools, TV, and trust: Introduction to the special issue on imitation in typically‐developing children. Journal of Experimental Child Psychology, 101, 225–227. doi:10.1016/j.jecp.2008.09.005 18930250 10.1016/j.jecp.2008.09.005

[cdev12549-bib-0011] Carruthers, P. (2002). Human creativity: Its cognitive basis, its evolution, and its connections with childhood pretence. The British Journal for the Philosophy of Science, 53, 225–249. doi:10.1093/bjps/53.2.225

[cdev12549-bib-0012] Chappell, J. , Cutting, N. , Tecwyn, E. C. , Apperly, I. A. , Beck, S. R. , & Thorpe, S. K. S. (2015). Minding the gap: A comparative approach to studying the development of innovation In Kaufman A. B. & Kaufman J. C. (Eds.), Animal creativity and innovation (pp. 287–314). London, UK: Academic Press.

[cdev12549-bib-0013] Cutting, N. , Apperly, I. A. , Chappell, J. , & Beck, S. R. (2014). The puzzling difficulty of tool innovation: Why can't children piece their knowledge together? Journal of Experimental Child Psychology, 125, 110–117. doi:10.1016/j.jecp.2013.11.010 24530037 10.1016/j.jecp.2013.11.010

[cdev12549-bib-0014] Dean, L. G. , Vale, G. L. , Laland, K. N. , Flynn, E. , & Kendal, R. L. (2013). Human cumulative culture: A comparative perspective. Biological Reviews, 89, 284–304. doi:10.1111/brv.12053 24033987 10.1111/brv.12053

[cdev12549-bib-0015] Defeyter, M. A. , Avons, S. E. , & German, T. C. (2007). Developmental changes in information central to artifact representation: Evidence from ‘functional fluency’ tasks. Developmental Science, 10, 538–546. doi:10.1111/j.1467‐7687.2007.00617.x 17683340 10.1111/j.1467-7687.2007.00617.x

[cdev12549-bib-0016] Defeyter, M. A. , & German, T. P. (2003). Acquiring an understanding of design: Evidence from children's insight problem solving. Cognition, 89, 133–155. doi:10.1016/S0010‐0277 12915298 10.1016/s0010-0277(03)00098-2

[cdev12549-bib-0017] Elias, S. (2012). Origins of human innovation and creativity: Breaking old paradigms In Elias S. (Ed.), Origins of human innovation and creativity (pp. 1–13). Oxford, UK: Developments in Quaternary Science, Elsevier.

[cdev12549-bib-0018] Enquist, M. , & Ghirlanda, S. (2007). Evolution of social learning does not explain the origin of human cumulative culture. Journal of Theoretical Biology, 246, 129–135. doi:10.1016/j.jtbi.2006.12.022 17275852 10.1016/j.jtbi.2006.12.022

[cdev12549-bib-0019] Flynn, E. , & Smith, K. (2012). Investigating the mechanisms of cultural acquisition: How pervasive is overimitation in adults? Social Psychology, 43, 185–195. doi:10.1027/1864‐9335/a000119

[cdev12549-bib-0020] Flynn, E. , & Whiten, A. (2012). Experimental “microcultures” in young children: Identifying biographic, cognitive, and social predictors of information transmission. Child Development, 83, 911–925. doi:10.1111/j.1467‐8624.2012.01747.x 22417384 10.1111/j.1467-8624.2012.01747.x

[cdev12549-bib-0021] Gopnik, A. (2012). Scientific thinking in young children: Theoretical advances, empirical research, and policy implications. Science, 337, 1623–1627. doi:10.1126/science.1223416 23019643 10.1126/science.1223416

[cdev12549-bib-0022] Griffin, A. S. , Lermite, F. , Perea, M. , & Guez, D. (2013). To innovate or not: Contrasting effects of social groupings on safe and risky foraging in Indian mynahs. Animal Behaviour, 86, 1291–1300. doi:10.1016/j.anbehav.2013.09.035

[cdev12549-bib-0023] Hanus, D. , Mendes, N. , Tennie, C. , & Call, J. (2011). Comparing the performances of apes (*Gorilla gorilla, Pan troglodytes, Pongo pygmaeus*) and human children (*Homo sapiens*) in the floating peanut task. PLoS ONE, 6, e19555. doi:10.1371/journal.pone.0019555 21687710 10.1371/journal.pone.0019555 PMC3110613

[cdev12549-bib-0024] Hauser, M. D. (2003). To innovate or not to innovate? That is the question In Reader S. M. & Laland K. N. (Eds.), Animal innovation (pp. 329–338). Oxford, UK: Oxford University Press.

[cdev12549-bib-0025] Hayes, B. K. , & Thompson, S. P. (2007). Causal relations and feature similarity in children's inductive reasoning. Journal of Experimental Psychology: General, 136, 470–484. doi:10.1037/0096‐3445.136.3.470 17696694 10.1037/0096-3445.136.3.470

[cdev12549-bib-0026] Heyes, C. (2012). What's social about social learning? Journal of Comparative Psychology, 126, 193–202. doi:10.1037/a0025180 21895355 10.1037/a0025180

[cdev12549-bib-0027] Horner, V. , & Whiten, A. (2005). Causal knowledge and imitation/emulation switching in chimpanzees (*Pan troglodytes*) and children (*Homo sapiens*). Animal Cognition, 8, 164–181. doi:10.1007/s10071‐004‐0239‐6 15549502 10.1007/s10071-004-0239-6

[cdev12549-bib-0028] Kacelnik, A. (2009). Tools for thought or thoughts for tools? Proceedings of the National Academy of Sciences of the United States of America, 106, 10071–10072. doi:10.1073/pnas.0904735106 19541623 10.1073/pnas.0904735106 PMC2700916

[cdev12549-bib-0029] Kandler, A. , & Laland, K. N. (2009). An investigation of the relationship between innovation and cultural diversity. Theoretical Population Biology, 76, 59–67. doi:10.1016/j.tpb.2009.04.004 19393256 10.1016/j.tpb.2009.04.004

[cdev12549-bib-0030] Kendal, R. L. , Coe, R. L. , & Laland, K. N. (2005). Age differences in neophilia, exploration, and innovation in family groups of callitrichid monkeys. American Journal of Primatology, 66, 167–188. doi:10.1002/ajp.20136 15940712 10.1002/ajp.20136

[cdev12549-bib-0100] Kendal, R. L. , Coolen, I. , van Bergen, Y. , & Laland, K. N. (2005). Tradeoffs in the adaptive use of social and asocial learning. Advances in the Study of Behavior, 35, 333–379. doi:10.1016/S0065‐3454(05)35008‐X.

[cdev12549-bib-0031] Kendal, J. , Giraldeau, L.‐A. , & Laland, K. (2009). The evolution of social learning rules: Payoff‐biased and frequency‐dependent biased transmission. Journal of Theoretical Biology, 260, 210–219. doi:10.1016/j.jtbi.2009.05.029 19501102 10.1016/j.jtbi.2009.05.029

[cdev12549-bib-0032] Kummer, H. , & Goodall, J. (1985). Conditions of innovative behavior in primates. Philosophical Transactions of the Royal Society B, 308, 203–214. doi:10.1098/rstb.1985.0020

[cdev12549-bib-0033] Laland, K. N. (2004). Social learning strategies. Learning & Behavior, 32(1), 4–14. 15161136 10.3758/bf03196002

[cdev12549-bib-0034] Laland, K. N. , & Janik, V. M. (2006). The animal cultures debate. Trends in Ecology and Evolution, 21, 542–547. 16806574 10.1016/j.tree.2006.06.005

[cdev12549-bib-0035] Laland, K. N. , & Reader, S. M. (2010). Comparative perspectives on human innovation In O'Brien M. J. & Shennan S. J. (Eds.), Innovation in cultural systems: Contributions from evolutionary anthropology (pp. 37–51). Cambridge, MA: MIT Press.

[cdev12549-bib-0036] Laland, K. N. , & Williams, K. (1998). Social transmission of maladaptive information in the guppy. Behavioral Ecology, 9, 493–499. doi:10.1093/beheco/9.5.493

[cdev12549-bib-0037] Lehmann, L. , Feldman, M. W. , & Kaeuffer, R. (2010). Cumulative cultural dynamics and the coevolution of cultural innovation and transmission: An ESS model for panmictic and structured populations. Journal of Evolutionary Biology, 23, 2356–2369. doi:10.1111/j.1420‐9101.2010.02096.x 20825551 10.1111/j.1420-9101.2010.02096.x

[cdev12549-bib-0038] Levitt, T. (1963). Creativity is not enough. Harvard Business Review, May/June, 72–83.

[cdev12549-bib-0039] Lewis, H. M. , & Laland, K. N. (2012). Transmission fidelity is the key to the build‐up of cumulative culture. Philosophical Transactions of the Royal Society B, 367, 2171–2180. doi:10.1098/rstb.2012.0119 10.1098/rstb.2012.0119 PMC3385684 22734060

[cdev12549-bib-0040] Mellars, P. (2005). The impossible coincidence. A single‐species model for the origins of modern human behavior in Europe. Evolutionary Anthropology, 14, 12–27.

[cdev12549-bib-0041] Mesoudi, A. , Laland, K. N. , Boyd, R. , Buchanan, B. , Flynn, E. , McCauley, R. N. , … Tennie, C. (2013). The cultural evolution of technology and science In Richerson P. J. & Christiansen M. H. (Eds.), Cultural evolution: Society, technology, language, and religion (pp. 193–216). Cambridge, MA: MIT Press.

[cdev12549-bib-0042] Nielsen, M. (2012). Imitation, pretend play, and childhood: Essential elements in the evolution of human culture? Journal of Comparative Psychology, 126, 170–181. 21859186 10.1037/a0025168

[cdev12549-bib-0043] Nielsen, M. (2013). Young children's imitative and innovative behaviour on the floating object task. Infant and Child Development, 22, 44–52. doi:10.1002/icd.1765

[cdev12549-bib-0044] Nielsen, M. , Tomaselli, K. , Mushin, I. , & Whiten, A. (2014). Exploring tool innovation: A comparison of Western and Bushman children. Journal of Experimental Child Psychology, 126, 384–394. doi:10.1016/j.jecp.2014.05.008 25014272 10.1016/j.jecp.2014.05.008

[cdev12549-bib-0045] O'Brien, M. J. , & Shennan, S. J. (2010). Issues in anthropological studies of innovation In O'Brien M. J. & Shennan S. J. (Eds.), Innovation in cultural systems: Contributions from evolutionary anthropology (pp. 3–17). Cambridge, MA: MIT Press.

[cdev12549-bib-0046] Overington, S. E. , Morand‐Ferron, J. , Boogert, N. J. , & Lefebvre, L. (2009). Technical innovations drive the relationship between innovativeness and residual brain size in birds. Animal Behaviour, 78, 1001–1010. doi:10.1016/j.anbehav.2009.06.033

[cdev12549-bib-0047] Picciuto, E. , & Carruthers, P. (2012). The origins of creativity In Paul E. S. & Kaufman S. B. (Eds.), The philosophy of creativity (pp. 199–223). Oxford, UK: Oxford University Press.

[cdev12549-bib-0048] Ramsey, G. , Bastian, M. L. , & van Schaik, C. (2007). Animal innovation defined and operationalized. Behavioral and Brain Sciences, 30, 393–437. doi:10.1017/S0140525X07002373 18081967 10.1017/S0140525X07002373

[cdev12549-bib-0049] Reader, S. M. , Hager, Y. , & Laland, K. N. (2011). The evolution of primate general and cultural intelligence. Philosophical Transactions of the Royal Society B, 366, 1017–1027. doi:10.1098/rstb.2010.0342 10.1098/rstb.2010.0342 PMC3049098 21357224

[cdev12549-bib-0050] Reader, S. M. , & Laland, K. N. (2002). Social intelligence, innovation, and enhanced brain size in primates. Proceedings of the National Academy of Sciences of the United States of America, 99, 4436–4441. doi:10.1073/pnas.062041299 11891325 10.1073/pnas.062041299 PMC123666

[cdev12549-bib-0051] Reader, S. M. , & Laland, K. N. (2003). Animal innovation: an introduction In Reader S. M. & Laland K. N. (Eds.), Animal innovation (pp. 3–35). Oxford, UK: Oxford University Press.

[cdev12549-bib-0052] Rendell, L. , Fogarty, L. , Hoppitt, W. J. E. , Morgan, T. J. H. , Webster, M. M. , & Laland, K. N. (2011). Cognitive culture: Theoretical and empirical insights into social learning strategies. Trends in Cognitive Sciences, 15, 68–76. doi:10.1016/j.tics.2010.12.002 21215677 10.1016/j.tics.2010.12.002

[cdev12549-bib-0053] Rendell, L. , Hoppitt, W. , & Kendal, J. (2007). Is all learning innovation? Behavioral and Brain Sciences, 30, 421–422. doi:10.1017/S0140525X07002373

[cdev12549-bib-0054] Runco, M. A. , & Acar, S. (2012). Divergent thinking as an indicator of creative potential. Creativity Research Journal, 24, 66–75. doi:10.1080/10400419.2012.652929

[cdev12549-bib-0055] Shennan, S. (2001). Demography and cultural innovation: A model and its implications for the emergence of modern human culture. Cambridge Archaeological Journal, 11, 5–16.

[cdev12549-bib-0056] Simonton, D. K. (2000). Creative development as acquired expertise: Theoretical issues and an empirical test. Developmental Review, 20, 283–318. doi:10.1006/drev.1999.0504

[cdev12549-bib-0057] Simonton, D. K. (2003). Human creativity: Two Darwinian analyses In Reader S. M. & Laland K. N. (Eds.), Animal innovation (pp. 309–325). Oxford, UK: Oxford University Press.

[cdev12549-bib-0058] Slater, P. J. B. , & Lachlan, R. F. (2003). Is innovation in bird song adaptive? In Reader S. M. & Laland K. N. (Eds.), Animal innovation (pp. 117–136). Oxford, UK: Oxford University Press.

[cdev12549-bib-0059] Sol, D. , Griffin, A. S. , & Bartomeus, I. (2012). Consumer and motor innovation in the common myna: The role of motivation and emotional responses. Animal Behaviour, 83, 179–188. doi:10.1016/j.anbehav.2011.10.024

[cdev12549-bib-0060] Sol, D. , Griffin, A. S. , Bartomeus, I. , & Boyce, H. (2011). Exploring or avoiding novel food resources? The novelty conflict in an invasive bird. PLoS ONE, 6, e19535. doi:10.1371/journal.pone.0019535 21611168 10.1371/journal.pone.0019535 PMC3097186

[cdev12549-bib-0061] Tecwyn, E. C. , Thorpe, S. K. S. , & Chappell, J. (2014). Development of planning in 4‐ to 10‐year‐old children: Reducing inhibitory demands does not improve performance. Journal of Experimental Child Psychology, 125, 85–101. doi:10.1016/j.jecp.2014.02.006 24858446 10.1016/j.jecp.2014.02.006

[cdev12549-bib-0062] Tennie, C. , Call, J. , & Tomasello, M. (2009). Ratcheting up the ratchet: On the evolution of cumulative culture. Philosophical Transactions of the Royal Society B, 364, 2405–2415. doi:10.1098/rstb.2009.0052 10.1098/rstb.2009.0052 PMC2865079 19620111

[cdev12549-bib-0063] Thornton, A. , & Samson, J. (2012). Innovative problem solving in wild meerkats. Animal Behaviour, 83, 1459–1468. doi:10.1016/j.anbehav.2012.03.018

[cdev12549-bib-0064] Thorpe, W. H. (1964). Learning and instinct in animals. London, UK: Methuen.

[cdev12549-bib-0065] Toelch, U. , Bruce, M. J. , Meeus, M. T. H. , & Reader, S. M. (2011). Social performance cues induce behavioral flexibility in humans. Frontiers in Psychology, 2, 1–7. doi:10.3389/fpsyg.2011.00160 21811477 10.3389/fpsyg.2011.00160 PMC3139953

[cdev12549-bib-0066] Vaesen, K. (2012). The cognitive bases of human tool use. Behavioral and Brain Sciences, 35, 203–262. doi:10.1017/S0140525X11001452 22697258 10.1017/S0140525X11001452

[cdev12549-bib-0067] van Schaik, C. P. , van Noordwijk, M. A. , & Wich, S. A. (2006). Innovation in wild Bornean orangutans (*Pongo pygmaeus wurmbii*). Behaviour, 143, 839–876. doi:10.1163/156853906778017944

[cdev12549-bib-0068] Walker, C. M. , & Gopnik, A. (2014). Toddlers infer higher‐order relational principles in causal learning. Psychological Science, 25, 161–169. doi:10.1177/0956797613502983 24270464 10.1177/0956797613502983

[cdev12549-bib-0069] Want, S. C. , & Harris, P. L. (2002). How do children ape? Applying concepts from the study of non‐human primates to the developmental study of ‘imitation’ in children. Developmental Science, 5, 1–14. doi:10.1111/1467‐7687.00194

[cdev12549-bib-0070] Whiten, A. , & Flynn, E. (2010). The transmission and evolution of experimental microcultures in groups of young children. Developmental Psychology, 46, 1694–1709. doi:10.1037/a0020786 20822212 10.1037/a0020786

[cdev12549-bib-0071] Whiten, A. , & van Schaik, C. P. (2007). The evolution of animal ‘cultures’ and social intelligence. Philosophical Transactions of the Royal Society B, 362, 603–620. doi:10.1098/rstb.2006.1998 10.1098/rstb.2006.1998 PMC2346520 17255007

[cdev12549-bib-0072] Wood, L. A. , Kendal, R. L. , & Flynn, E. G. (2013). Copy me or copy you? The effect of prior experience on social learning. Cognition, 127, 203–213. doi:10.1016/j.cognition.2013.01.002 23454793 10.1016/j.cognition.2013.01.002

